# An Efficient Synthesis and Preliminary Investigation of Novel 1,3-Dihydro-*2H*-benzimidazol-2-one Nitro and Nitramino Derivatives

**DOI:** 10.3390/ma15238330

**Published:** 2022-11-23

**Authors:** Jonas Šarlauskas, Jonita Stankevičiūtė, Jelena Tamulienė

**Affiliations:** 1 Life Sciences Centre, Department of Xenobiotic Biochemistry, Institute of Biochemistry, Vilnius University, Sauletekio av. 7, 01513 Vilnius, Lithuania; 2 Life Sciences Centre, Department of Molecular Microbiology and Biotechnology, Institute of Biochemistry, Vilnius University, Sauletekio av. 7, 01513 Vilnius, Lithuania; 3 Physics Faculty, Institute of Theoretical Physics and Astronomy, Vilnius University, Sauletekio av. 3, 01513 Vilnius, Lithuania

**Keywords:** 1,3-dihydro-*2H*-benzimidazol-2-one, nitration, nitrocompounds, nitramines, thermal analysis (TGA), thermostable high energy materials, synthesis, X-ray diffraction, properties

## Abstract

The preparation and properties of a series of novel 1,3-dihydro-*2H*-benzimidazol-2-one nitro and nitramino derivatives are described. A detailed crystal structure of one of the obtained compounds, 4,5,6-trinitro-1,3-dihydro-*2H*-benzimidazol-2-one (TriNBO), was characterized using low temperature single crystal X-ray diffraction, namely an orthorhombic yellow prism, space group ‘P 2 21 21′, experimental crystal density 1.767 g/cm^3^ (at 173 K). Methyl analog, 5-Me-TriNBO-monoclinic red plates, space group, P 21/c, crystal density 1.82 g/cm^3^. TriNBO contains one activated nitro group at the fifth position, which was used for the nucleophilic substitution in the aminolysis reactions with three monoalkylamines (R=CH_3_, C_2_H_5_, (CH_2_)_2_CH_3_) and ethanolamine. The 5-R-aminoderivatives were further nitrated with N_2_O_5_/ HNO_3_ and resulted in a new group of appropriate nitramines: 1,3-dihydro-*2H*-5-R-N(NO_2_)-4,6-dinitrobenzimidazol-2-ones. Thermal analysis (TGA) of three selected representatives was performed. The new compounds possess a high melting point (200–315 °C) and thermal stability and can find a potential application as new thermostable energetic materials. Some calculated preliminary energetic characteristics show that TriNBO, 5-Me-TriNBO, 5-methylnitramino-1,3-dihydro-*2H*-4,6-dinitrobenzimidazol-2-one, and 5-nitratoethylnitramino-1,3-dihydro-*2H*-4,6-dinitrobenzimidazol-2-one possess increased energetic characteristics in comparison with TNT and tetryl. The proposed nitrocompounds may find potential applications as thermostable high-energy materials.

## 1. Introduction

Various types of nitrocompounds contain appropriate structural fragments: (a) C-NO_2_; (b) O-NO_2_, and (c) N-NO_2_ which are differentiated into three groups (nitrocompounds, organic nitrates, and nitramines) [1]. The representatives of the latter group, organic nitramines, are widely used as high-energy materials (HEMs) and components of explosive compositions, propellants, or solid rocket fuel [2,3]. Heterocyclic nitroderivatives containing a nitramino group have been insufficiently investigated when compared to benzene analogs (tetryl, pentryl, etc.) and alicyclic (RDX, HMX, CL-20, and some others that are similar) nitramine compounds, nowadays produced in the industry in bulk quantities [4,5]. Some representatives of pyridine nitramines have been investigated in the published work of Polish scientists [6,7,8]. A few 1,2,4-triazole and tetrazole nitramines have been mostly studied during the last decade by researchers originating from Germany [9,10,11] and Russia [12,13]. An interesting work dedicated to energetic nitroimidazole nitramines has recently been published by USA authors [14].

**Figure 1:**
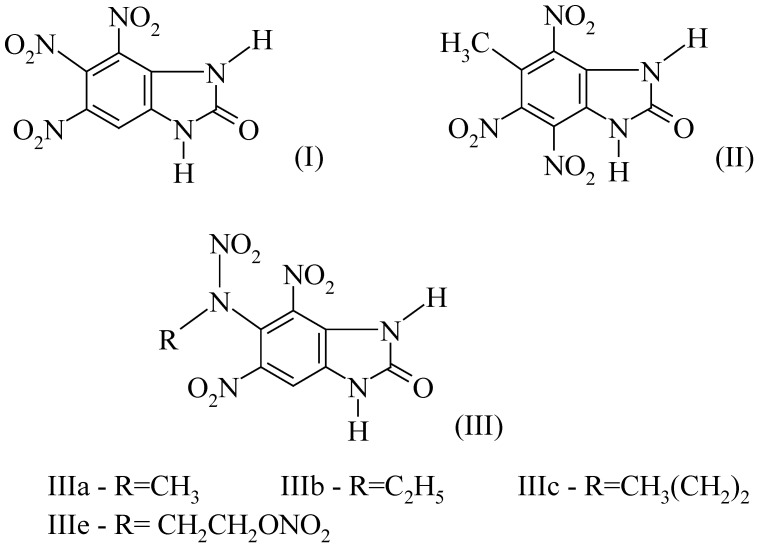
Structures of prepared 1,3-dihydro-*2H*-benzimidazol-2-one nitro and nitramino derivatives.

In continuation to our previous studies [15,16,17,18], herein we describe the synthesis, LC-MS analysis, X-ray structure, and preliminary investigation of properties of some new nitro and nitramino benzimidazole derivatives. Most of the nitration procedures, which have been previously applied for the nitration of benzimidazole derivatives [19,20,21,22,23,24,25,26,27,28,29,30], were carried out in a step-by-step procedure, introducing one nitro group in one reaction. In the present work we successfully introduced three nitro groups in one synthetic procedure by our modified efficient nitration methodology (Figure 1).

## 2. Experimental

### 2.1. General Methods and Materials

All chemical reagents and solvents were purchased from commercial suppliers (Sigma-Aldrich (St. Louis, MO, USA), TCI-Europe (Zwijdrecht, Belgium) and Merck (Darmstadt, Germany)) and were used without purification unless otherwise specified. The N_2_O_5_/HNO_3_ solution was prepared from HNO_3_ and P_2_O_5_ according to the known method [31]. The purity of the compounds was monitored by TLC, using silica gel 60 F254 aluminum plates (Merck, Darm-stadt, Germany). Visualization was performed under UV light. The flash column chromatography was carried out by using a Wakogel C-200 (Wako Chemical, Osaka, Japan) silica gel. The calculated densities and other molecular characteristics of the investigated compounds (3–6) were obtained using ACD Labs software (Toronto), vers.11. The melting points were determined in open capillaries using a MEL-TEMP apparatus (Barn-stead Thermolyne Corp., Dubuque, IA, USA). UV-VIS spectra were recorded using a Lambda 25 UV-VIS spectrophotometer (PerkinElmer, Waltham, MA, USA). IR spectra were recorded in KBr on a PerkinElmer spectrophotometer (FT-IR Spectrum BX II). NMR spectra were obtained with Bruker spectrometer (400 MHz) in d6-dimethylsulfoxide, using a residual solvent signal as an internal standard. Liquid chromatography-mass spectrometry (LC–MS) analyses were performed using an HPLC system (CBM-20A controller, two LC-2020AD pumps, SIL-30AC autosampler, and CTO-20AC column oven; Shimadzu, Japan) equipped with photodiode array (PDA) detector (SPD-M20A Prominence diode array detector; Shimadzu, Japan) and mass spectrometer (LCMS-2020, Shimadzu, Japan) equipped with an ESI source. The chromatographic separation was conducted using a Hydrosphere C18 column, 4 × 150 mm^2^ (YMC, Kyoto, Japan) at 40 °C and a mobile phase that consisted of 0.1% formic acid (solvent A) and acetonitrile (solvent B) delivered in gradient elution mode at a flow rate of 0.6 mL min^−1^. The elution program that was used was as follows: isocratic 0% B for 0.5 min, from 0 to 60% B over 4.5 min, isocratic 60% B for 0.1 min, from 60 to 0% B over 0.1 min, isocratic 0% B for 5 min. Mass scans were measured from *m*/*z* 10 up to 1000, at 350 °C interface temperature, 250 °C DL temperature, ±4500 V interface voltage, and neutral DL/Qarray, using N_2_ as nebulizing and drying gas. Mass spectrometry data were acquired in both the positive and the negative ionization modes. The data were analyzed using Lab-Solutions LC-MS software (YMC, Japan). Thermogravimetric analysis was performed on a PerkinElmer thermogravimetric analyzer, STA6000 TGA/DSC. Density measurements were performed at 293 K using an AccuPyc 1330 Pycnometer (Micromeritics) Single crystal X-ray diffraction was evaluated using the instrument “Bruker-Nonius KappaCCD” (Computing data collection ‘KappaCCD’) and computing data reduction “Denzo and Scalepak (Otwinowski and Minor, 1997)” were applied. All diagrams and calculations were performed using maXus (Bruker Nonius, Delft & MacScience, Tokyo, Japan).

### 2.2. Synthesis and Properties Investigation of 1,3-Dihydro-2H-benzimidazol-2-one Polynitrocompounds

Trinitrosubstituted 1,3-dihydro-*2H*-benzimidazol-2-one derivatives were prepared by the application of a convenient and efficient one-pot nitration reaction, which avoids the use of an aggressive fuming nitric acid and multistep procedures.

#### 2.2.1. Synthesis of 4,5,6-Trinitro-1,3-Dihydro-*2H*-benzimidazol-2-one (TriNBO) (**1**) in One Step

**Scheme 1:**
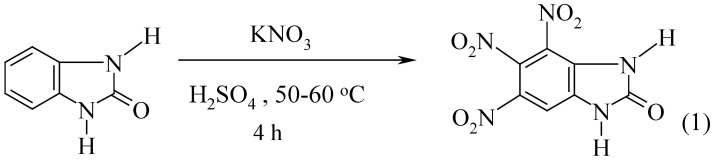
Nitration reaction of 1,3-dihydro-*2H*-benzimidazol-2-one for the one step preparation of TriNBO (**1**).

**Figure 2:**
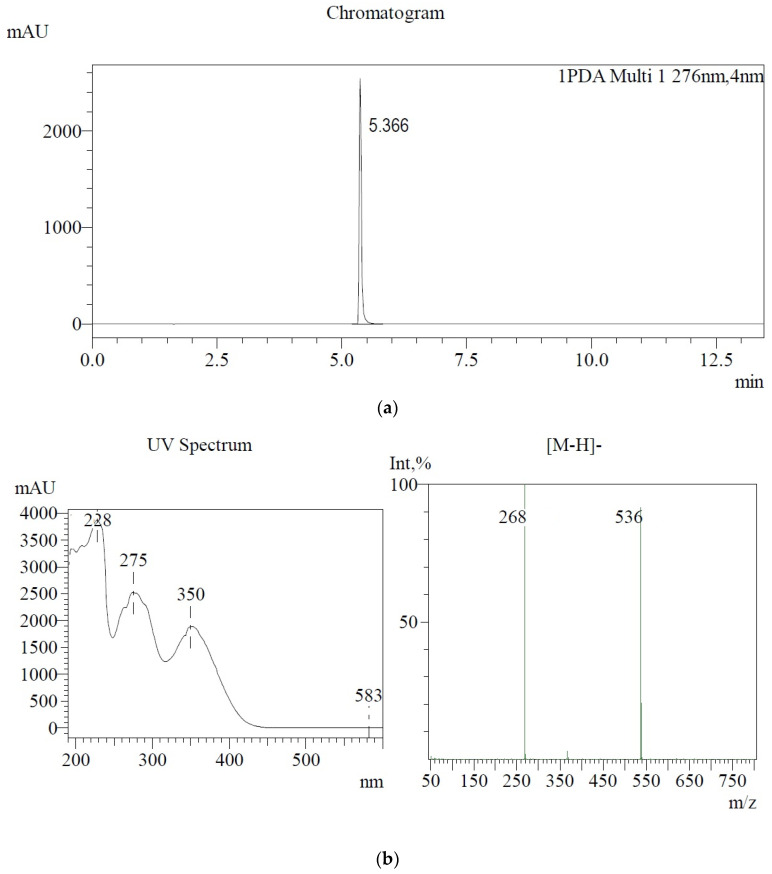
(**a**,**b**) LC-MS analysis data of TriNBO, obtained by nitration of 1,3-dihydro-*2H*-benzimidazol-2-one.

The main compound (TriNBO) (**1**) was prepared from 1,3-dihydro-*2H*-benzimidazol -2-one, by nitration reaction using an efficient system derived from potassium nitrate/sulphuric acid:

A total of 13.4 g (0.1 M) of 1,3-dihydro-*2H*-benzimidazol-2-one was added in small portions by stirring and cooling (ice/water) to the 33.3 g (0.33 mol) KNO_3_, solution in 100 mL H_2_SO_4_ (98%) (Scheme 1 below). When the whole amount of the compound was added, the reaction mixture was slowly (during 1 h) warmed up to 60 °C. Then, the reaction mixture was stirred at 50–60 °C for 4 h. Finally, the reaction mixture was allowed to cool to room temperature and then cooled to 4 °C in the freezer (12 h). The obtained yellow crystalline product was filtered off on the porous glass filter and washed several times with 50% cold H_2_SO_4_, then with cold distilled water and dried at 100 °C overnight. The yield was 22.3 g (83%), m. p. 314–315 °C (sublimation with decomp.) (lit. m.p. 313–315 °C (dec.) [32]. The obtained reaction product (TriNBO) was pure and did not contain any impurities of dinitro- or tetranitro-derivatives (LC-MS analysis data are presented below in the next section in Figure 2a,b).

#### 2.2.2. LC-MS Analysis of the Reaction Product, 4,5,6-Trinitro-1,3-Dihydro-*2H*-benzimidazol-2-one (TriNBO) (**1**)

A sample of the nitration reaction product (2 mg) was dissolved in 1 mL of DMSO and analyzed by LC-MS. The analysis results are presented in Figure 2a,b below.

Analysis data: spectrum UV_max_: 228, 275, and 350 nm; the main TriNBO molecular ion in negative mode: [M-H]^−^ = 268. A typical dimeric negative ion at 536 was also observed.

The obtained analysis results clearly demonstrate that nitration reaction product is pure trinitroderivative, TriNBO, which does not contain any dinitro- or tetranitro- derivatives impurities.

Additionally, the main properties of this material were described.

#### 2.2.3. The Main Physicochemical Properties of 4,5,6-Trinitro-1,3-Dihydro-*2H*-benzimidazol-2-one (TriNBO)

The main properties of TriNBO (C_7_H_3_N_5_O_7_) are shortly represented below.

TriNBO in its amorphous or microcrystalline state is a stable yellow powder, almost insoluble in water and concentrated sulfuric acid, but is better soluble in nitric acid. With alcohols (especially methanol) and some other polar solvents, TriNBO forms stable solvates. TriNBO is well soluble in DMSO, DMF, dimethylacetamide, sulfolane, methyl acetate, methanol, nitromethane, and nitrobenzene. It is much less soluble in benzene, toluene, and xylene and poorly soluble in other solvents such as acetone, hexane, dichloroethane, or CCl_4_. TriNBO is stable during storage at room temperature. The analysis results of a sample in our laboratory did not show any changes even after 20 years.

#### 2.2.4. Spectral Properties of TriNBO

**Figure 3:**
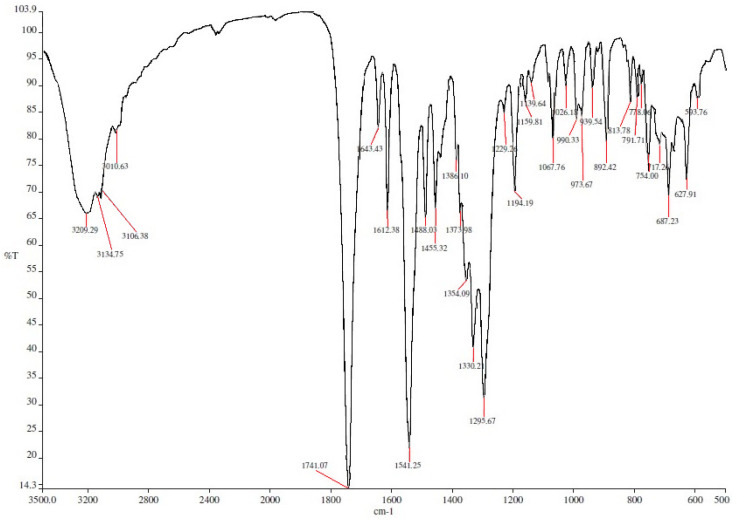
FT−IR spectrum of TriNBO (KBr).

FT-IR spectrum of TriNBO, registered in KBr disc, is shown in Figure 3. The main characteristic peaks are NH (3209), CO (1741), and NO_2_ (1541, 1330) (cm^−1^).

Full IR spectrum (cm^−1^): 3209, 1741, 1643, 1612, 1541, 1488, 1455, 1354, 1330, 1296, 1229, 1194, 1067, 990, 974, 892, 813, 792, 754, 687, 628, 594.

NMR spectra: ^1^H spectrum (d6-DMSO, 400MHz): 12.66 (s, 1H, 3-NH). 12.29 (s. 1H, 1-NH) 7.96 (s, 1H, 7-H-Ar). ^13^C spectrum (d6-DMSO, 100 MHz): 155.75 (C-2); 134.52 (C-6), 134.08 (C-5); 132.83 (C-8); 131.73 (C-9); 122.56 (C-4); 107.82 (C7).

#### 2.2.5. Thermal Properties Investigation

**Figure 4:**
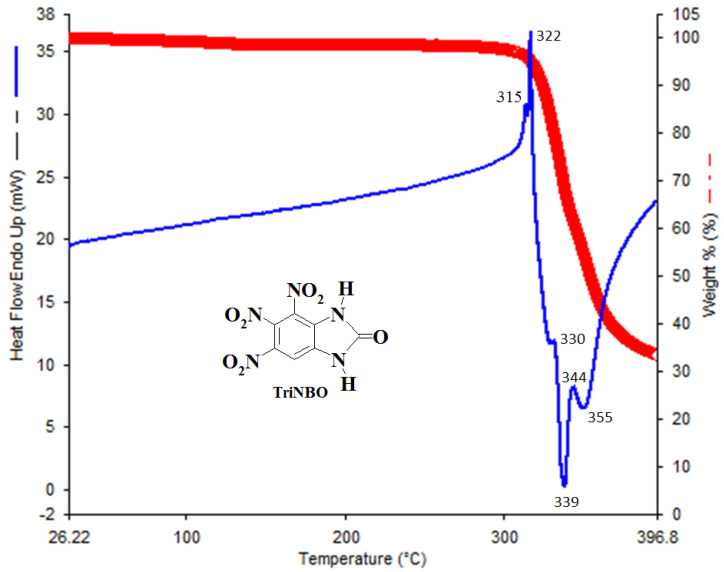
Thermogravimetric analysis of 4,5,6-trinitro-1,3-dihydro-*2H*-benzimidazol-2-one (TriNBO) (**1**) sample (5 mg) (5 °C/min).

Analysis of the thermal properties of TriNBO was performed on a PerkinElmer thermogravimetric analyzer, STA6000 TGA/DSC at 5 °C/min (Figure 4):

Thermal degradation of TriNBO is a complex process. There are two separate processes that are observed simultaneously on heating, partial sublimation, and decomposition after starting mass loss at the point of 315 °C. The total mass loss reached a maximum of 339 °C (decomposition without deflagration).

#### 2.2.6. Crystal Properties of TriNBO (X-ray Diffraction Structure Analysis)

**Figure 5:**
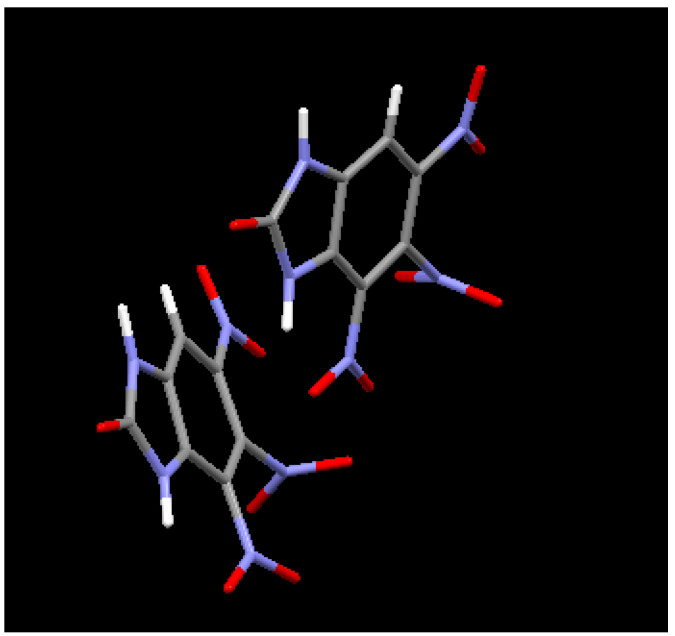
3D-TriNBO-crystal cell packing image that was obtained from the Mercury program (illustrated hydrogen bonding possibility among neighbor –NH- and -NO_2_ functional groups). Hydrogen bonding is an important factor of the high density of this trinitrocompound.

**Figure 6:**
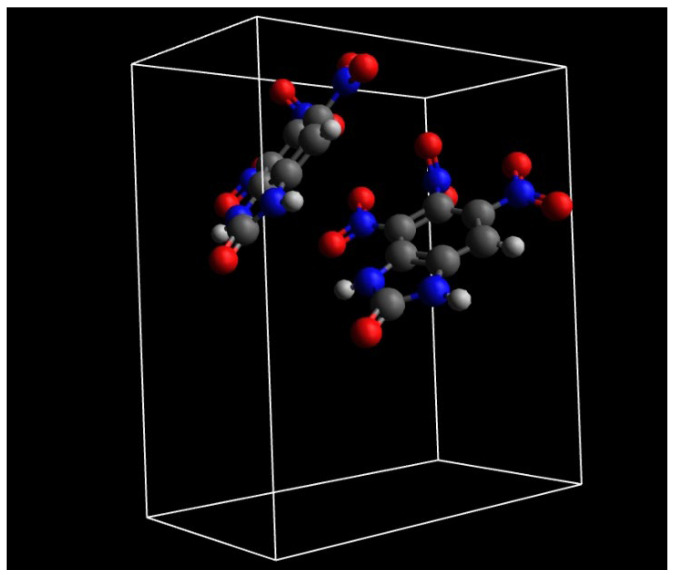
TriNBO-crystal cell unit packing illustration shows the dimeric mode of two TriNBO molecules with a slightly different space rearrangement.

**Figure 7:**
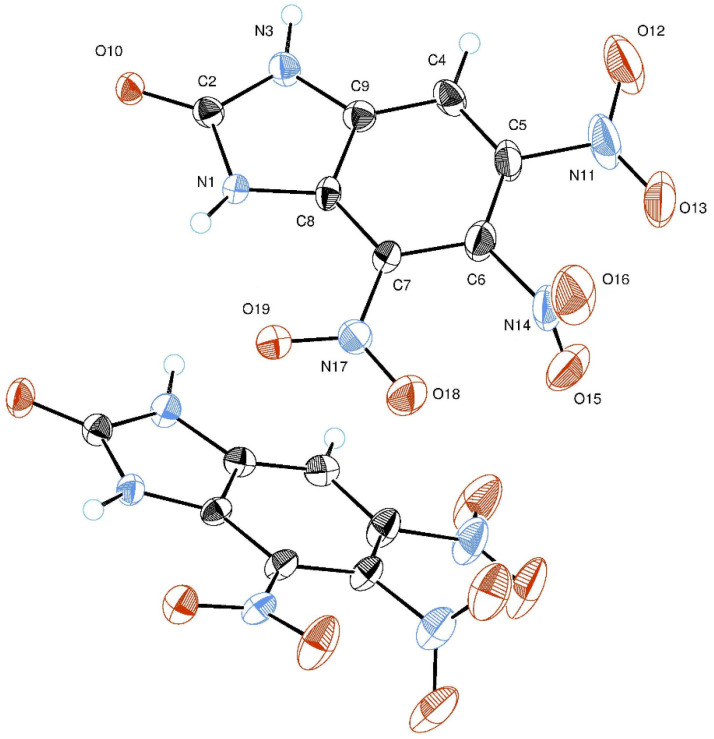
Ortep image at the 50% probability of thermal ellipsoids for (TriNBO)—(the crystal was obtained by growing from the cold solution in 95% HNO_3_). (CCDC: 1882607).

This was undertaken to establish 4,5,6-trinitro-1,3-dihydro-*2H*-benzimidazol-2-one three-dimensional structure and density. The crystals of TriNBO (yellow prisms) for its X-ray diffraction were obtained by long-standing in the freezer (at −18 °C temperature) of its solution in 95% HNO_3_ (the reason for the selection of such non-ordinary solvent was to avoid solvate formation). The geometries of TriNBO are tabulated below (Figure 5, Figure 6 and Figure 7). The main crystallographic data are shown below in Table 1.

The TriNBO (4,5,6-trinitro-1,3-dihydro-*2H*-benzimidazol-2-one) crystal cell (volume: 2023.12(16) Å^3^), containing two molecules of the nitrocompound is represented in Figure 5 and Figure 6.

An Ortep image of the TriNBO X-Ray structure is shown in Figure 7.

**Figure A1:**
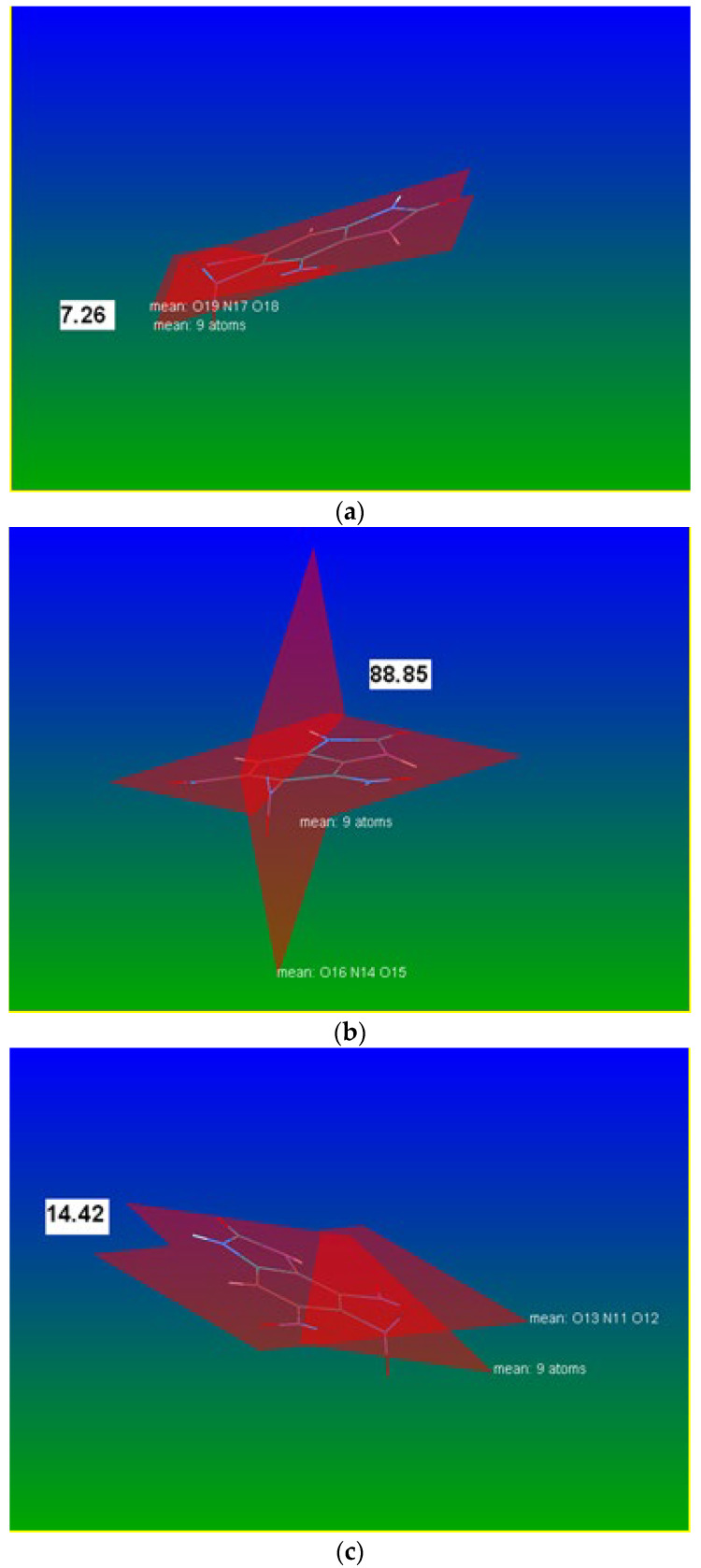
(**a**–**c**) Schematic representation of dihedral angles between 4, 5, and 6 nitrogroups and benzimidazol-2-one plane (values of angles for nitrogroups: 4-NO_2_: 7.26° 5-NO_2_: 88.85° and for 6-NO_2_: 14.42°). (**a**) 4-NO_2_. (**b**) 5-NO_2_. (**c**) 6-NO_2_.

A detailed list of bond and angles values and other X-ray data are placed in Appendix A and Appendix B (Table A1 and Figure A1). Schematic representation of dihedral angles is shown in Figure A1 in Appendix B.

The crystal structure investigation results clearly demonstrate that dihedral angles between 4, 5, and 6 nitro groups and benzimidazol-2-one plane are very different (angles for nitrogroups: 4-NO_2_: 7.26° 5-NO_2_: 88.85° and for 6-NO_2_: 14.42°) and the longest nitrogroup bond C-N (1.495 Å) is for 5-NO_2_ (activated nitro group, which can participate in substitution reactions). Other neighbor nitro group C-N bonds are substantially shorter: 4-NO_2_ (1.427 Å) and 6-NO_2_ (1.471 Å). We explored the activity of the 5-NO_2_ group in TriNBO for the derivatization and synthesis of the new energetic nitramines, possessing 4,6-dinitrobenzimidazole moiety.

#### 2.2.7. Preparation of 5-Methyl Analog of TriNBO (Heterocyclic Analog of TNT), 5-Methyl-4,6,7-trinitro-1,3-Dihydro-*2H*-benzimidazol-2-one (5-Me-TriNBO) (**2**)

The starting compound 5-methyl-1,3-dihydro-*2H*-benzimidazol-2-one, was obtained by the published method by heating a mixture of 4-methyl-1,2-phenylenediamine and urea at 180 °C [33].

**Scheme 2:**
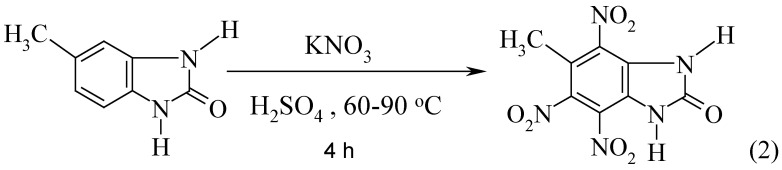
Efficient nitration reaction of 5-methyl-1,3-dihydro-*2H*-benzimidazol-2-one for the preparation of 5-methyl-TriNBO (**2**) in one step.

The 5-Me-TriNBO was synthesized using a convenient one-step nitration reaction of 5-methyl-1,3-dihydro-*2H*- benzimidazol-2-one, by the application of the same nitration system derived from potassium nitrate/sulphuric acid, but in modified temperature conditions (Scheme 2 below):

KNO_3_ totaling 33.3 g (0.33 mol) was added in small portions to 100 mL of conc. H_2_SO_4_ (98%) with stirring and cooling (5 °C, ice-water bath) and the homogenous nitration mixture was obtained. This was followed by the careful addition of 14.82 g (0.1 mol) 5-methyl-1,3-dihydro-*2H*-benzimidazol- 2-one in small portions until the reaction mixture became a clear light yellow. Hereafter, the reaction temperature was gradually raised to about 60 °C for 2 h, and finally, the mixture was heated to 90 °C for 4 h. The resulting mixture was allowed to cool to room temperature and then transferred to a closed vessel and placed in the freezer (+4 °C) to stand overnight. The obtained orange-red microcrystalline supernatant was filtered using sintered glass filter. The product was washed with 50% H_2_SO_4_ and then several times with distilled H_2_O and finally dried at 100 °C to the constant weight. The yield was 25.4 g, 89.7%. M.p. 285-7 °C (dec.). The spectral data are summarized in Table A2 in Appendix C. The physicochemical properties (solubility, and others) of 5-methyl-1,3-dihydro-*2H*-benzimidazol- 2-one are almost similar to TriNBO, however, 5-methyl group insertion in aromatic ring leads to the substantial decrease in thermostability. The obtained one step reaction product (5-Me-TriNBO) was pure and did not contain any dinitro-derivative impurities (LC-MS analysis data are represented below).

#### 2.2.8. LC-MS-Analysis Data for 5-Me-TriNBO (5-Methyl-4,6,7-trinitro-1,3-dihydro-*2H*-benzimidazol-2-one (**2**))

**Figure 8:**
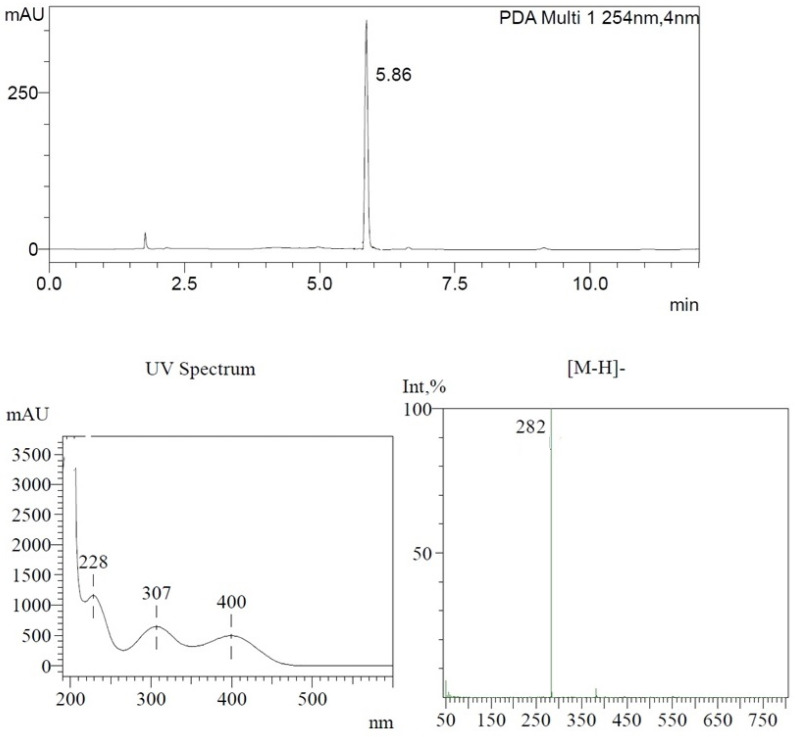
LC-MS analysis of 5-methyl-1,3-dihydro-*2H*-benzimidazol-2-one nitration product (2 mg/1 mL DMSO), 5-methyl-4,6,7-trinitro-1,3-dihydro-*2H*-benzimidazol-2-one (5-Me-TriNBO, (**2**)). UV spectrum λ_max_ = 228, 307, 400 nm; R.T.= 5.86 min, molecular ion: [M-H]^−^ = 282 in negative mode.

The product of the nitration reaction of 5-methyl-1,3-dihydro-*2H*-benzimidazol-2-one, 2 mg 5-Me-TriNBO solution in 1 mL dimethyl sulfoxide was used for LC-MS analysis. The results are shown in Figure 8.

#### 2.2.9. Crystal Properties of 5-Me-TriNBO (**2**), 5-Methyl-4,6,7-trinitro-1,3-dihydro-*2H*-benzimidazol-2-one (X-ray Diffraction Structure Analysis)

**Figure 9:**
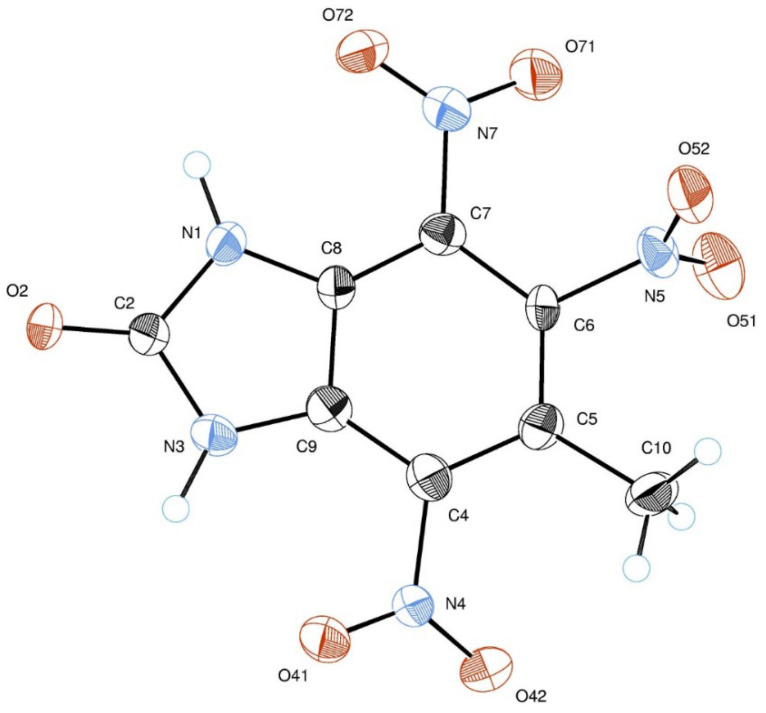
Ortep image at the 50% probability of thermal ellipsoids for 5-methyl-4,6,7-trinitro-1,3-dihydro-*2H*-benzimidazol-2-one (5-Me-TriNBO, (**2**))—(crystal (red plate)) was obtained from the solution in nitromethane.

This was undertaken to establish the 5-methyl-4,5,6-trinitro-1,3-dihydro-*2H*-benzimidazol-2-one three-dimensional structure and density. The crystals of 5-Me-TriNBO (red plates) for its X-ray diffraction were obtained by prolonged standing in the freezer (at +4 °C temperature) of the compound solution in nitromethane. The Ortep of the 5-MeTriNBO is shown below (Figure 9) and the main crystallographic data are represented in Table 2.

#### 2.2.10. TriNBO Structure Functionalization for the Synthesis of New Energetic Benzimidazole Nitramines

Structure analysis revealed that the 5-NO_2_ group, with a maximal angle (88.85°) and increased bond (N-C) length (1.495 angstroms) can demonstrate a heightened chemical reactivity and especially increased reactivity in various displacement reactions of this NO_2_ group with various basic amino-compounds [34]. The starting 5-NH(R)-4,6-dinitrobenzimidazolones (R=CH_3_, C_2_H_5_ CH_3_(CH_2_)_2_, and HOCH_2_CH_2_) were prepared by a simple aminolysis procedure that was described in our previous work by the reaction in ethanol (70 °C, 8 h) of TriNBO with an appropriate amine [34].

#### 2.2.11. General Procedure for the Preparation of 5-R-Substituted Nitramines of 4,6-Dinitro-1,3-dihydro-*2H*-benzimidazol-2-one (**3**–**6**)

The nitramine derivatives of benzimidazol-2-one were prepared by nitration of previously published 5-NHR-substituted 4,6-dinitro-1,3-dihydro-*2H*-benzimidazol- 2-ones [34], by the application of an efficient nitration system: 40% N_2_O_5_/HNO_3_ in methylene chloride medium. This system was found to be more effective and cleaner then previously used nitric and sulphuric acid mixtures [35,36,37] for the nitramine synthesis by *N*-nitration.

**Scheme 3:**
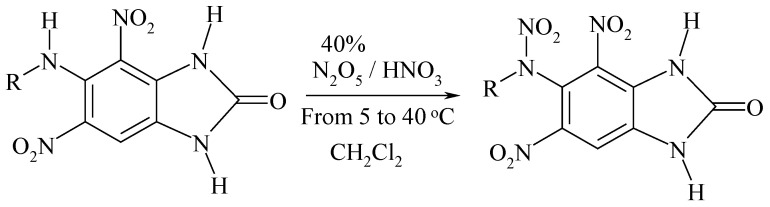
General scheme for the preparation of 5-R-nitramines of 4,6-dinitro-1,3-dihydro-*2H*-benzimidazol-2-one (where R=CH_3_ (**3**), CH_3_CH_2_ (**4**), CH_3_CH_2_CH_2_ (**5**), and O_2_NOCH_2_CH_2_ (**6**)).

By stirring and cooling (ice/ water bath) (0.01 mol) of appropriate 5-NHR 1,3-dihydro-*2H*-benzimidazol-2-one was added in small portions to 40% N_2_O_5_/ HNO_3_ solution (5 mL) in 100 mL CH_2_Cl_2_. (Scheme 3 below). When the whole compound was added, the reaction mixture was slowly (during 1 h) warmed up to 40 °C. Then, the reaction mixture was stirred with a magnetic stirrer for an additional 4 h and allowed to stand at room temperature overnight. Finally, the reaction mixture was stirred and cooled to 0–5 °C in crushed ice, and the residual nitric acid was carefully neutralized by the addition of 15 g powdered MgCO_3_. The obtained neutralized yellow solution of nitramine was filtered off on the porous glass filter, and the solid supernatant of magnesium salts were washed several times with CH_2_Cl_2_ and the solvent was removed on a rotary evaporator. The resulting yellow crystalline product was recrystallized from 70% acetic acid and dried at 60 °C.

The obtained N-nitration reaction products, nitramines, were substantially pure and did not contain any other nitroderivative impurities (the LC-MS analysis data are presented in Table 3 in the next section).

The yields of synthesis, m.p., and spectral data of the prepared nitramines are presented in Table 3.

#### 2.2.12. Thermal Analysis of Selected Nitramines

There were two representatives of synthesized nitramine compounds (5-MeN(NO_2_)DNBO (**3**) and 5-O_2_NO(CH_2_)_2_N(NO_2_)DNBO (**6**)) that possess more interesting energetic characteristics, so, it was important to investigate their thermostability.

**Figure 10:**
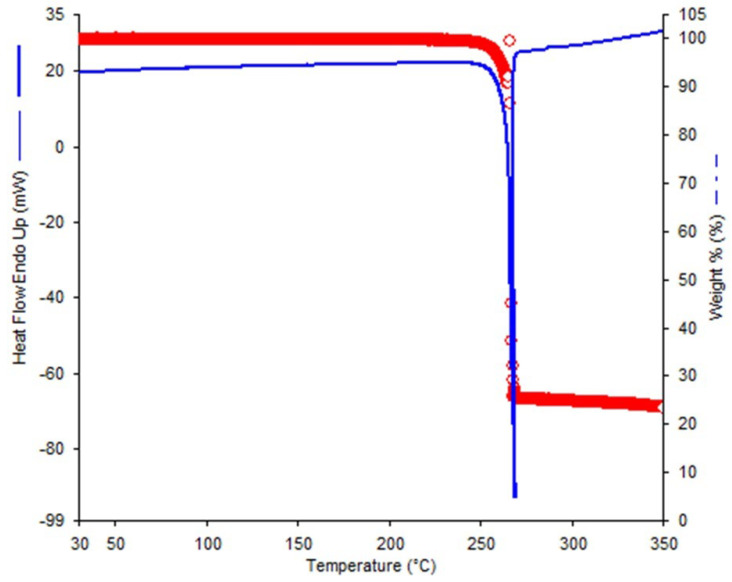
Termogravimetric analysis of 5-MeN(NO_2_)DNBO (**3**), 5-methylnitramino-4,6-dinitro-1,3-dihydrobenzimidazol-2-one at 5 °C/min. (DSC, blue line; TGA, red line).

**Figure 11:**
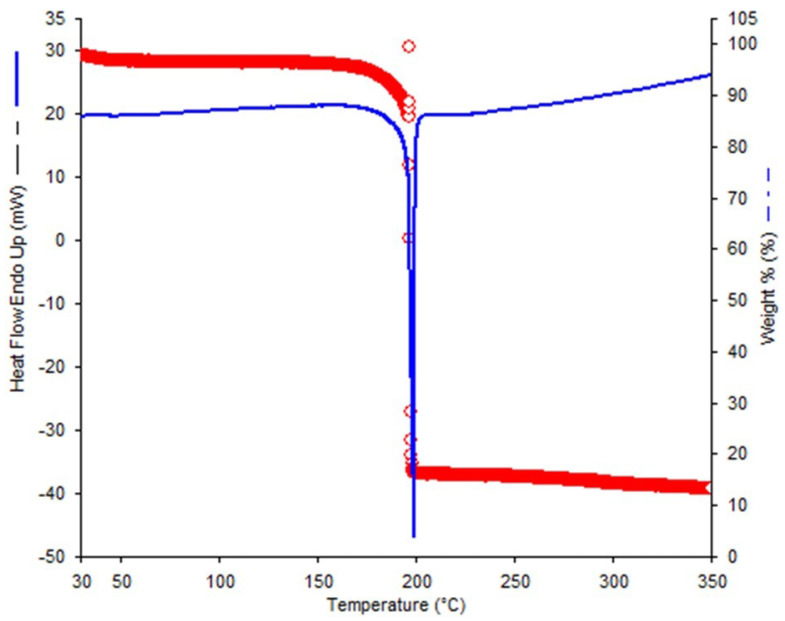
Thermogravimetric analysis of (**6**), 5-[2′-(Nitroxyethyl)nitramino]-4,6-dinitro-1,3-dihydrobenzimidazol-2-one at 5 °C/min.

The thermal stability of synthesized compounds was investigated at 5 °C/min. The results of the thermogravimetric analysis are represented in Figure 10 and Figure 11 below.

The above thermal analysis (TGA, red line; DSC, blue line, deflagration stage is marked as red bubbles line) data clearly show that organic nitrato group (O_2_NOCH_2_CH_2_-) strongly decreases the decomposition temperature in the case of 5-[2′-(nitroxyethyl)nitramino]-4,6-dinitro-1,3-dihydro-*2H*-benzimidazol-2-one (deflagration at 199–201 °C) when compared to 5-methylnitramino-4,6-dinitro-1,3-dihydro-*2H*-benzimidazol-2-one (deflagration at 278 °C). Importantly, the thermal stability of both nitramine derivatives are substantially diminished in comparison with benzimidazol-2-one trinitro derivative TriNBO (**1**) (decomposition at 339 °C).

#### 2.2.13. The Main Physicochemical and Spectral Properties of Synthesized 1,3-Dihydro-*2H*-benzimidazol-2-one Nitrocompounds and Nitramines

The nitration reaction yields and the main properties of the synthesized new nitrocompounds are summarized in Table 3 and Table A2 in Appendix C:

## 3. Results and Discussion

### Calculated Detonation Performance of Benzimidazol-2-one Nitro and Nitramino Derivatives

The preliminary data on the energetic properties and detonating performance of newly synthesized benzimidazol-2-one nitro and nitramino compounds are presented in Table 4.

The present data were calculated by applying the appropriate formulas presented below:

Velocity of detonation (m/s) [38]:

D = −393.6877 − 0.2454(NE/M) − 114.0793 (E/M).

where N is the number of NO_2_ groups, E denotes the total energy of the derivative under study, and M is molar mass.

Detonation pressure [39]:

P(kbar)= 15.58 (D ρ/(1.01(1 + 1.30 ρ))^2^

where ρ is the density of the compounds.

These obtained data demonstrate that the calculated detonation velocity (D) and detonation pressure of most of the new derivatives are superior to the known standard compound TNT, however, their characteristics in general are something less when compared with other standard compound tetryl characteristics.

## 4. Conclusions

TriNBO (4,5,6-trinitro-1,3-dihydro-*2H*-benzimidazol-2-one) and 5-Me-TriNBO (5-methyl-4,6,7-trinitro-1,3-dihydro-*2H*-benzimidazol-2-one) have been synthesized in 83 and 90% yields in a one-step procedure of efficient nitration of benzimidazol-2-one and 5-methylbenzimidazol-2-one with KNO_3_/H_2_SO_4_.

The crystal structure and experimental crystal densities of TriNBO (1.76 g/cm^3^) and 5-Me-TriNBO (1.82 g/cm^3^) were investigated. We revealed that TriNBO crystallizes as orthorhombic yellow prisms in the space group P 2 21 21, while its methyl analog, 5-Me-TriNBO as monoclinic red plates in the space group P 21/c.

The results of thermal analysis (DTA) of TriNBO showed onset at 315 °C and the highest degree of decomposition at 339 °C, which means that the thermostability of TriNBO is superior to that of HNS (hexanitrostilbene), widely known as thermostable HEM [8,13,14].

The main detonation characteristics (velocity of detonation and detonation pressure) of the new nitrocompounds were calculated. The calculated detonation velocity and detonation pressure of TriNBO, its methyl analog, 5-Me-TriNBO and benzimidazol-2-one nitramines are superior to the experimental parameters of the standard aromatic energetic trinitrocompound TNT, and in general they are about equal to the detonation parameters of standard tetranitrocompound tetryl.

Our prepared energetic nitramines, 5-CH_3_N(NO_2_)DNBO) and 5-(ONO_2_CH_2_CH_2_-N(NO_2_)DNBO possess good energetic characteristics, but their thermostability is substantially lower in comparison with that of TriNBO. Nitramines containing N-ethyl and N-propyl groups are substantially less thermostable and possess slightly worse energetic properties because of their diminished oxygen balance.

## Figures and Tables

**Table 1:** The main crystallographic data for TriNBO (**1**).

Empirical formula	C_7_H_3_N_5_O_7_
Formula weight	269.14
Temperature	173(2) K (−100 °C)
Wavelength	0.71073 Å
Crystal system	Orthorhombic
Crystal description	Yellow prism
Space group	P 2 21 21
Unit cell dimensions	a = 7.3420(5)
A alpha = 90.0 deg.	
b = 16.1900(2) A beta = 90.0000(12) deg.	
c = 17.0200(6) A gamma = 90.0 deg.	
Volume	2023.12(16) Å^3^
Z	8
Density (calculated)	1.767 g/cm^3^
Density (measured, pycnometer)	1.76 g/cm^3^
Absorption coefficient	0.161 mm^−1^
F(000)	1088
Crystal size	0.27 × 0.22 × 0.15 mm
Two theta max. for data	28.0 deg.
Reflections collected	9135
Independent reflections	4800
Refinement method	Full-matrix least-squares on F_2_
R indices (all data)	R_1_ = 0.1010, wR_2_ = 0.2141

**Table 2:** The main crystallographic data for 5-MeTriNBO (**2**).

Empirical formula	C_8_H_5_N_5_O_7_
Formula weight	283.17
Temperature	193(2) K (−80.15 °C)
Wavelength	0.71073 Å
Crystal system	Monoclinic
Crystal description	Red plate
Space group	P 21/c
Unit cell dimensions	a = 4.8952(5)
A alpha = 90.0 deg.	
b = 14.7707(18) A beta = 93.253(6) deg.	
14.3037(19) A gamma = 90.0 deg.	
Volume	1032.6(2) Å^3^
Z	4
Density (calculated, (−80 °C) from X-Ray data)	1.822 g/cm^3^
Density (measured, (20 °C) pycnometer)	1.78 g/cm^3^
Absorption coefficient	0.163 mm^−1^
F(000)	576
Crystal size	0.33 × 0.212 × 0.02 mm
Refinement method	Full-matrix least-squares on F_2_
Measurement device	‘Bruker–Nonius KappaCCD’

**Table 3:** The main data of the synthesized 1,3-dihydro-*2H*-benzimidazol-2-one nitro- and nitramino compounds.

No.	Abbreviation and Chemical Name	Mol. Formula	Mol. Weight	LC-MS Data	Yield, (%)	M.p. (°C)
1	TriNBO, 4,5,6-Trinitro-1,3-dihydro- benzimidazol-2-one	C_7_H_3_N_5_O_7_	269.13	R.T. = 5.37 min, UV, λ_max_ = 275, 350. [M-H]^−^ =268 2[M-H]^−^ = 536.	83.0	314-5 (dec.)
2	5-Me-TriNBO, 5-Methyl-4,6,7-trinitro-1,3-dihydrobenzimidazol-2-one	C_8_H_5_N_5_O_7_	283.16	R.T. = 5.86 min, UV λ_max_ = 307, 400. [M-H]^−^ =282	89.8	270-2 (dec.)
3	5-MeN(NO_2_)DNBO, 5-Methylnitramino-4,6-dinitro-1,3-dihydrobenzimidazol-2-one	C_8_H_6_N_6_O_7_	298.13	R.T. = 5.58 min, UV λ_max_ = 279, 351. [M-H]^−^ = 297 [2M-H]^−^ = 595.	85.0	278-9 (deflagr.)
4	5-EtN(NO_2_)DNBO, 5-Ethylnitramino-4,6-dinitro-1,3-dihydrobenzimidazol-2-one	C_9_H_8_N_6_O_7_	312.20	R.T. = 5.61min, UV λ_max_ = 280, 353. [M-H]^−^ = 311 [2M-H]^−^ = 623.	87.3	271-3 (dec.)
5	5-PrN(NO_2_)DNBO, 5-Propylnitramino-4,6-dinitro-1,3-dihydrobenzimidazol-2-one	C_10_H_10_N_6_O_7_	329.23	R.T. = 5.37min, UV λ_max_ = 275, 350. [M-H]^−^ = 326 [2M-H]^−^ = 653.	78.1	261-3 (dec.)
6	5-O_2_NO(CH_2_)_2_N(NO_2_) DNBO, 5-[2′-(Nitroxyethyl)nitramino]- 4,6-dinitro-1,3-dihydrobenzimidazol-2-one	C_9_H_7_N_7_O_10_	373.20	R.T. = 5.37min, UV λ_max_ = 275, 350. [M-H]^−^ = 372 [2M-H]^−^ = 545 [M-NO_2_]^−^ = 326	73.0	199-201 (deflagr.)

**Table 4:** The main calculated energetic properties of the synthesized 1,3-dihydro-*2H*-benzimidazol-2-one trinitroderivatives.

No.	Compound	Molecular Formula	Calc. Density (g/cm^3^)	Oxygen Balance, (Ω), OB_CO2_, %	Velocity of Detonation, Calc. (D, (m/s))	Detonation Pressure, Calc., P (kbar)
1.	TriNBO	C_7_H_3_N_5_O_7_	1.767 (at173K temp.) or 1.76 (exp., pycnom.)	−50.52%	7900	284
2.	5-Me-TriNBO	C_8_H_5_N_5_O_7_	1.822 (at193 K temp.) or 1.82 (exp., pycnom.)	−64.98	7460	245
3.	5-MeN(NO_2_)DNBO	C_8_H_6_N_6_O_7_	1.806	−64.39	7370	240
4.	5-EtN(NO_2_)DNBO	C_9_H_8_N_6_O_7_	1.719	−76.87	6980	209
5.	5-PrN(NO_2_)DNBO	C_10_H_10_N_6_O_7_	1.647	−87.47	6800	194
6.	5-O_2_NO(CH_2_)_2_N(NO_2_)DNBO	C_9_H_7_N_7_O_10_	1.859	−68.78	7570	253
7.	TNT (as standard)	C_7_H_5_N_3_O_6_	1.630 (exp.)	−73.98	6900	210 (exp.)
8.	Tetryl (as standard)	C_7_H_5_N_5_O_8_	1.73 (exp.)	−47.40	7570	260 (at 1.71g/cm^3^) (exp.)

## Data Availability

Samples of the compounds are not available from the authors but can be specially prepared under request.

## Appendix A

**Table A1:** Selected bond lenths (A) and angles (B) for the compound TriNBO obtained from X-ray diffraction data analysis.

(A) Intramolecular bond lengths	
O19 N17 1.224(6)	O10A C2A 1.236(6)
N1 C8 1.380(6)	N14A O16A 1.197(8)
N1 C2 1.395(6)	N14A O15A 1.226(7)
N1 H1 0.9519	N14A C6A 1.474(7)
O10 C2 1.229(6)	O19A N17A 1.226(5)
C9 N3 1.383(7)	N17A O18A 1.211(6)
C9 C4 1.375(7)	N17A C7A 1.470(6)
C9 C8 1.420(7)	C9A C4A 1.373(7)
N17 O18 1.204(6)	C9A N3A 1.383(6)
N17 C7 1.427(7)	C9A C8A 1.422(6)
N3 C2 1.368(6)	C7A C8A 1.390(7)
N3 H3 0.9600	C7A C6A 1.400(7)
C8 C7 1.379(7)	C4A C5A 1.387(7)
C7 C6 1.424(7)	C4A H4A 0.9600
C4 C5 1.372(8)	N1A C8A 1.355(6)
C4 H4 0.9600	N1A C2A 1.377(6)
C6 C5 1.400(8)	N1A H1A 0.9599
C6 N14 1.495(7)	N3A C2A 1.385(6)
N14 O16 1.235(9)	N3A H3A 1.0112
N14 O15 1.222(9)	O12A N11A 1.184(7)
C5 N11 1.471(7)	C5A C6A 1.401(7)
N11 O13 1.202(8)	C5A N11A 1.493(7)
N11 O12 1.233(8)	N11A O13A 1.202(7)
(B) Intramolecular bond angles	
C8 N1 C2 109.3(4)	O16A N14A O15A 124.6(6)
C8 N1 H1 121.4	O16A N14A C6A 118.8(5)
C2 N1 H1 126.5	O15A N14A C6A 116.6(5)
N3 C9 C4 131.8(4)	O19A N17A O18A 123.8(4)
N3 C9 C8 107.1(4)	O19A N17A C7A 117.1(4)
C4 C9 C8 121.0(4)	O18A N17A C7A 118.9(4)
O18 N17 O19 123.3(4)	C4A C9A N3A 131.0(4)
O18 N17 C7 120.4(4)	C4A C9A C8A 122.6(4)
O19 N17 C7 116.3(4)	N3A C9A C8A 106.4(4)
C9 N3 C2 109.7(4)	C8A C7A C6A 120.3(4)
C9 N3 H3 119.8 9	C8A C7A N17A 117.6(4)
C2 N3 H3 130.4	C6A C7A N17A 122.0(4)
O10 C2 N3 127.6(4)	C9A C4A C5A 117.1(4)
O10 C2 N1 125.2(4)	C9A C4A H4A 120.5
N3 C2 N1 107.2(4)	C5A C4A H4A 122.4
N1 C8 C7 131.8(4)	C8A N1A C2A 109.5(4)
N1 C8 C9 106.7(4)	C8A N1A H1A 130.5
C7 C8 C9 121.5(4)	C2A N1A H1A 120.0
C8 C7 N17 121.2(4)	C9A N3A C2A 108.8(4)
C8 C7 C6 117.3(4)	C9A N3A H3A 116.4
N17 C7 C6 121.4(4)	C2A N3A H3A 134.5
C5 C4 C9 117.4(4)	O10A C2A N1A 125.8(4)
C5 C4 H4 122.6	O10A C2A N3A 126.8(4)
C9 C4 H4 120.1	N1A C2A N3A 107.4(4)
C5 C6 C7 119.1(5)	C4A C5A C6A 122.8(5)
C5 C6 N14 121.2(5)	C4A C5A N11A 116.6(4)
C7 C6 N14 119.7(4)	C6A C5A N11A 120.6(5)
O16 N14 O15 129.3(6)	C7A C6A C5A 118.6(5)
O16 N14 C6 115.0(6)	C7A C6A N14A 120.1(4)
O15 N14 C6 115.7(6)	C5A C6A N14A 121.3(5)
C4 C5 C6 123.4(5)	O13A N11A O12A 125.2(5)
C4 C5 N11 116.8(5)	O13A N11A C5A 118.5(5)
C6 C5 N11 119.8(5)	O12A N11A C5A 116.2(5)
O13 N11 O12 122.9(6)	N1A C8A C7A 133.8(4)
O13 N11 C5 121.1(5)	N1A C8A C9A 107.8(4)
O12 N11 C5 115.9(6)	C7A C8A C9A 118.3(4)

## Appendix C

**Table A2:** Spectral data of synthesized 1,3-dihydro-*2H*-benzimidazol-2-one nitrocompounds.

No.	Compound	Molecular Formula	FT-IR Spectra, (cm^−1^)	^1^H NMR Spectra, (400 Mz, d6-DMSO), ppm
1.	TriNBO	C_7_H_3_N_5_O_7_	3209, 1741, 1643, 1612, 1541, 1488, 1455, 1354, 1330, 1296, 1229, 1194, 1067, 990, 974, 892, 813, 792, 754, 687, 628, 594.	12.66 (s, 1H, 3-NH). 12.29 (s. 1H, 1-NH) 7.96 (s, 1H, 7-H-Ar).
2.	5-Me-TriNBO	C_8_H_5_N_5_O_7_	3173, 1737, 1607, 1551, 1424, 1390, 1358, 1332, 1300, 1202, 1075, 1042, 994, 941, 908, 818, 786, 758, 701, 681, 601.	12.51 (s. 1H, 3-NH). 12.44 (s. 1H, 1-NH), 2.33 (s. 3H, CH_3_).
3.	5-MeN(NO_2_)DNBO	C_8_H_6_N_6_O_7_	3191, 3015, 1742, 1642, 1613, 1539, 1509, 1488, 1436, 1340, 1307, 1196, 1061, 1017, 988, 943, 899, 822, 804, 784, 754, 722, 685, 632, 593.	12.41 (s. 1H, 3-NH). 12.12 (s. 1H, 1-NH) 7.98 (s, 1H, 7-H-Ar), 3.60 (s, 3H, N-CH_3_)).
4.	5-EtN(NO_2_)DNBO	C_9_H_8_N_6_O_7_	3209, 1741, 1643, 1612, 1541, 1488, 1455, 1447, 1386, 1374, 1354, 1330, 1296, 1229, 1194, 1160, 1139, 1068, 1026, 990, 974, 939, 892, 814, 792, 778, 754, 717, 687, 665, 628, 594, 501.	12.39 (s. 1H, 3-NH). 12.12 (s. 1H, 1-NH) 7.96 (s. 1H, 7-H-Ar), 4.02 (m. *2H*, N-CH_2_), 1.06 (m. 3H).
5.	5-PrN(NO_2_)DNBO	C_10_H_10_N_6_O_7_	3215, 3021, 2964, 2932, 2876, 1742, 1642, 1612, 1541, 1488, 1457, 1438, 1384, 1352, 1329, 1313, 1286, 1195, 1072, 1028, 990, 952, 908, 895, 807, 754, 725, 686, 628, 603.	12.26 (s. *2H*, 1-NH, 3-NH) 7.96 (s, 1H, 7-H-Ar), 3.85 (m, *2H*, CH_2_), 1.45 (m. *2H*, CH_2_,), 0.82 ((m. 3H, CH_3_).
6.	5-O_2_NO(CH_2_)_2_N(NO_2_)DNBO	C_9_H_7_N_7_O_10_	3218, 1744, 1644, 1613, 1542, 1488, 1430, 1348, 1324, 1290, 1194, 1139, 1053, 1016, 989, 969, 933, 892, 837, 802, 781, 754, 718, 689, 631, 506.	12.32 (s. 1H, 3-NH). 12.11 (s. 1H, 1-NH) 7.95 (s. 1H, 7-H-Ar), 4.03 (m. 4H, N-CH_2_CH_2_)).

## References

[1] Agrawal J.P., Hodgson R. (2007). Organic Chemistry of Explosives.

[2] Pagoria P.F., Lee G.S., Mitchell A.R., Schmidt R.D. (2002). A review of energetic materials synthesis. Thermochim. Acta.

[3] Coburn M.D., Harris B.W., Lee K.Y., Stinecipher M.M., Heyden H.H. (1986). Explosives synthesis at Los Alamos. Ind. Eng. Chem. Prod. Res. Dev..

[4] Bagal L.I., Pevzner M.S., Frolov A.N., Sheludyakova N.I. (1970). Heterocyclic compounds. I. Synthesis of nitroderivatives of 1,2,4-triazole, 1,3,4-thiadiazole, tetrazole, 1,3,4-oxadiazole and pyrazole by uncatalitic replacement of diazogroup by nitrogroup. Chem. Heterocycl. Compd..

[5] Ostrovskii V.A., Pevzner M.S., Kofman T.P., Tselinskii I.V. (1999). Energetic 1,2,4-triazoles and tetrazoles: Synthesis, structure and properties. Targets Heterocycl. Syst..

[6] Klapötke T.M., Stierstorfer J. (2009). The CN7− Anion. J. Am. Chem. Soc..

[7] Urbanski T. (1964). Chemistry and Technology of Explosives.

[8] Urbanski T. (1964). Chemistry and Technology of Explosives.

[9] Stierstorfer J., Klapötke T.M., Hammerl A., Chapman R.D. (2008). 5-Azido-1H-tetrazole—Improved Synthesis, Crystal Structure and Sensitivity Data. Z. Anorg. Allg. Chem..

[10] Klapötke T.M., Sabaté C.M., Stierstorfer J. (2009). Neutral 5-nitrotetrazoles: Easy initiation with low pollution. New J. Chem..

[11] Klapötke T.M., Sabaté C.M., Stierstorfer J. (2008). Hydrogen-bonding Stabilization in Energetic Perchlorate Salts: 5-Amino-1H-tetrazolium Perchlorate and its Adduct with 5-Amino-1H-tetrazole. Z. Anorg. Allg. Chem..

[12] Koldobskii G.I., Soldatenko D.S., Gerasimova E.S., Khokhriakova N.R., Scherbinin M.B., Lebedev V.P., Ostrovskii V.A. (1997). Tetrazoles: Structure and properties of 5-nitrotetrazole. Zh. Org. Khim..

[13] Orlova Y.Y. (1973). Chemistry and Technology of High Explosives.

[14] Everest D. (2008). The Power of High Explosives in Theory and Practice.

[15] Anusevičius Ž., Soffers A.E.M.F., Čėnas N., Šarlauskas J., Segura-Aguilar J., Rietjens I.M.C.M. (1998). Quantitative structure-activity relationships for the conversion of nitrobenzimidazoles by DT-diaphorase: Implication for the kinetic mechanism. FEBS Lett..

[16] Anusevičius Ž., Soffers A.E.M.F., Čėnas N., Šarlauskas J., Martinez-Julvez M., Rietjens I.M.C.M. (1999). Quantitative structure-activity relationships for the electron transfer reactions of Anabaena PCC 7119 ferredoxin-NADP+ oxidoreductase with nitrobenzene and nitrobenzimidazolone derivatives: Mechanistic implications. FEBS Lett..

[17] Čėnas N., Nemeikaitė-Čėnienė A., Šarlauskas J., Anusevičius Ž., Nivinskas H., Misevičienė L., Marozienė A., Sunahara G.I., Lotufo G., Kuperman R.G., Hawari J. (2009). Mechanisms of the Mammalian Cell Cytotoxicity of Explosives, in Ecotoxicology of Explosives.

[18] Šarlauskas J., Dičkancaitė E., Nemeikaitė A., Anusevičius Ž., Nivinskas H., Segura-Aguilar J., Čėnas N. (1997). Nitrobenzimidazoles as substrates for DT-diaphorase and redox cycling compounds: Their enzymatic reactions and cytotoxicity. Archiv. Biochem. Biophys..

[19] Efros L.S., El‘tsov A.V. (1957). Investigation of benzimidazole. 15. Nitration of benzimidazolone and 1,3-dimethylbenzimidazolone. Zh. Obshch. Khim..

[20] Tselinskii I.V. (1997). Applications of energetic materials in engineering, technology and national economy. Soros Educat. J..

[21] Wright J.B. (1951). The chemistry of the benzimidazoles. Chem. Revs..

[22] Hofmann J.B. (1953). Imidazole and Its Derivatives.

[23] Preston N. (1974). Synthesis, reactions, and spectroscopic properties of benzimidazoles. Chem. Revs..

[24] Stefaniak L., Kamienski B., Voronkov M.G., Larina L.I., Lopyrev V.A., Webb G.A. (1991). Investigation of Benzimidazolones. Part 7: A 13C and 15N NMR Study of Some Nitro Benzimidazolones. Bull. Pol. Acad. Sci. Chem..

[25] Larina L., Lopyrev V. (2009). Topics in Applied Chemistry. Nitroazoles: Synthesis, Structure and Applications.

[26] Patai S. (1982). The Chemistry of Amino, Nitro and Nitroso Compounds.

[27] Benchidmi M., El Kihel A., Essassi E.M., Knouzi N., Toupet L., Danion-Baugot R., Carrie R. (1995). Nitration of substituted benzimidazoles. Bull. Soc. Chim. Belg..

[28] Freyer A.J., Lowema C.K., Nissan R.A., Wilson W.S. (1992). Synthesis and Explosive Properties of Dinitropicrylbenzimidazoles, and the ‘Trigger Linkage’ in Dinitropicrylbenzotriazoles. Austral. J. Chem..

[29] Grimmett M.R. (1997). Imidazole and Benzimidazole Synthesis (Best Synthetic Methods).

[30] Kaiya T., Nakamura K., Tanaka M., Miyata N., Kohda K. (2004). Product Analyses of Ozone Mediated Nitration of Benzimidazole Derivatives with Nitrogen Dioxide: Formation of 1-Nitrobenzimidazoles and Conversion to Benzotriazoles. Chem. Pharm. Bull..

[31] Holleman A.F., Wiberg E., Wiberg N. (2001). Inorganic Chemistry.

[32] Schindlbauer H., Kwiecinski W. (1976). Direct nitration of benzimidazolone and reaction of some of the nitration products. Monatsh. Chem..

[33] Clark R.L., Pessolano A.A. (1958). Synthesis of Some Substituted Benzimidazolones. J. Am. Chem. Soc..

[34] Šarlauskas J., Sergedienė E., Nemeikaitė-Čėnienė A., Nivinskas H., Ačaitė J., Čėnas N. (2000). Cytotoxicity of new bifunctional nitrobenzimidazoles. Acta Med. Litu..

[35] Clarkson C.E., Holden I.G., Malkin T. (1950). 322. The nitration of dimethylaniline to tetryl, 2: 4: 6: N-tetranitro-methylaniline. The course of the reaction. J. Chem. Soc..

[36] Naixing W., Boren C., Yuxiang O. (1994). Review on Benzofuroxan System Compounds. Propellants Explos. Pyrotech..

[37] Naixing W., Boren C., Yuxiang O. (1992). Synthesis of N,N’-bis-(2-nitrobenzodifuroxanyl)-3,5-dinitro-2,6-diaminopyridine. Propellants Explos. Pyrotech..

[38] Türker L. (2010). Velocity of detonation-a mathematical model. Acta Chim. Slov..

[39] Kamlet M.J., Jacobs S.J. (1968). Chemistry of Detonations. I. Simple Method for Calculating Detonation Properties of CHNO Explosives. J. Chem. Phys..

